# Physicochemical Properties, Polyphenol and Mineral Composition of Different Triticale Varieties Cultivated in the Republic of Moldova

**DOI:** 10.3390/molecules30061233

**Published:** 2025-03-10

**Authors:** Georgiana Gabriela Codină, Florin Ursachi, Adriana Dabija, Sergiu Paiu, Iurie Rumeus, Svetlana Leatamborg, Galina Lupascu, Silviu-Gabriel Stroe, Aliona Ghendov-Mosanu

**Affiliations:** 1 Faculty of Food Engineering, “Stefan cel Mare” University, 720229 Suceava, Romania; florin.ursachi@fia.usv.ro (F.U.); adriana.dabija@fia.usv.ro (A.D.); silvius@fia.usv.ro (S.-G.S.); 2 Faculty of Food Technology, Technical University of Moldova, 9/9 Studentilor St., MD-2045 Chisinau, Moldova; sergiu.paiu@doctorat.utm.md (S.P.); rumeus.iurie@usch.md (I.R.); aliona.mosanu@tpa.utm.md (A.G.-M.); 3 Faculty of Economics, Engineering and Applied Sciences, Cahul State University “Bogdan Petriceicu Hasdeu”, 1 Independence Square, MD-3909 Cahul, Moldova; 4 Applied Genetics Laboratory, Institute of Genetics, Physiology and Plant Protection, Moldova State University, 20 Padurii St., MD-2002 Chisinau, Moldova; svetlana.leatamborg@sti.usm.md (S.L.); galina.lupascu@sti.usm.md (G.L.)

**Keywords:** triticale composition, minerals, phenolic profile, FT-IR, principal component analysis

## Abstract

The quality characteristics of seven triticale grain varieties were determined by different physiochemical analyses. For this purpose, the content of protein, wet gluten, fat, ash, moisture, carbohydrates, test weight, and thousand-kernel mass; mineral elements Ca, Na, Zn, Fe, and Cu; and total phenolic content (TPC), total flavonoid content (TFC), free radical scavenging activity (DPPH assay), and phenolic profile (4-hydroxybenzoic acid, vanillic acid, caffeic acid, chlorogenic acid, *p*-coumaric acid, and rosmarinic acid) were analyzed. Also, Fourier transform infrared spectroscopy (FT-IR) was used to evaluate the quality parameters of triticale grains. According to the chemical data obtained, all triticale varieties may be used for breadmaking. A high variability was obtained among triticale varieties for mineral elements and antioxidant compounds. The highest values were recorded for Ca, followed by Fe, Na, Zn, and Cu. The TPC, TFC, DPPH, and phenolic compounds of the analyzed triticale samples increased with the increasing temperature used in the extraction method. Generally, the highest value for phenolic acid was obtained by *p*-coumaric acid followed by rosmarinic acid, caffeic acid, 4-hydroxybenzoic acid, vanillic acid, and chlorogenic acid. Principal component analysis of triticale cultivars related to their physicochemical data showed close association between some varieties such as Costel; Ingen 54, Ingen 35, Ingen 33, and Ingen 93, and Ingen 40; and Fanica varieties.

## 1. Introduction

Climate change, rapid population growth, and the current consumer preference for healthier foods have led to the search for alternative solutions to traditional cereals used in human nutrition. One such solution is triticale, which is the first cereal obtained from artificial hybridization between tetraploid wheat (*Triticum aestivum*) and rye (*Secale cereale*). In 2022, the top ten triticale producers in the world, by area cultivated and share, were Poland (34.1%), Belarus (11.2%), France (9.4%), Germany (9%), Spain (7.8%), China (5.5%), Turkey (2.8%), Lithuania (1.7%), Australia (1.7%), and Romania (1.6%), with more than 90.0% of triticale production being concentrated in Europe [1].

This non-traditional cereal has come to the attention of specialists through the study of the impact, importance, and uses of triticale in human nutrition [2]. The high capacity for environmental adaptation, the demand for health-promoting foods, as well as the need to grow cereals on marginal lands to feed a growing global population and more sustainable agriculture make triticale a cereal of the future [3].

The chemical composition and nutritional properties of triticale vary significantly due to the rather large number of genotypes [4]. The unique nutritional value of triticale grains is due to higher protein content (14–15 g/100 g) compared to wheat and rye, which has been inherited from its parents. Triticale proteins contain more essential amino acids, especially lysine (0.31–0.71 g/100 g), the first limiting amino acid in cereals—threonine, valine, leucine, etc.—and have better digestibility than wheat proteins [4,5,6]. Triticale has starch content (63.3–68.8 g/100 g dry weight) comparable to that of rye and wheat. It produces high levels of α-amylase, which can make starch more digestible. However, the ratio of amylopectin to amylose varies considerably depending on the variety. For example, in this cereal, the amylose content is highly variable, from 12.8 to 35.1 g/100 g of total starch, compared to the amylose content found in wheat, which can vary from 26.9 to 42.8 g/100 g. In terms of non-starch polysaccharide content, triticale has values much closer to wheat than to rye [4]. Triticale has a high fiber content (11.7–13.6 g/100 g), close to that of wheat, but with a higher quantity of water-extractable arabinoxylans, which give it viscous properties in aqueous solution [4,6].

Triticale flour also contains phenolic compounds with antioxidant activity, 5-alkylresorcinols, phytoestrogens, mineral substances (potassium, sodium, phosphorus, copper, manganese, iron, and calcium), and vitamins [7,8]. Triticale, with its rye ancestry, may exhibit different levels of polyphenols compared to wheat, potentially offering a unique health-promoting profile. The high quantity of antioxidants in triticale grains [9] contribute to health promotion, which is similar to many dietary supplements, nutraceuticals, and functional food ingredients found nowadays. From those, polyphenols are essential compounds that can be found in high amounts in triticale grains. Triticale grains contain phenolic acids such as ferulic acid, caffeic acid, p-coumaric acid, and vanillic acid. Among these, ferulic acid accounts for the highest amount, contributing significantly to the antioxidant activity of triticale grains [10]. Some authors have reported the presence of chlorogenic acid in some triticale varieties, though its amount was affected by processing techniques. However, the presence of other polyphenolic compounds such as vanillic acid can also be detected in triticale grains depending on the extraction conditions. For example, its value is very low or undetectable even at moderate temperature (40 °C), probably due to thermal degradation or volatile loss [11]. Because triticale contains significant concentrations of biological compounds with antioxidant activity, it is possible that it contributes to their daily intake, if they are present in an active form in triticale-based foods [12,13]. It is also worth noting that triticale grains are high in dietary fiber, of which the content exceeds 13%, which makes them effective as prebiotics in food products. Regarding the largest quantities of mineral elements, it has been reported that triticale grains contain calcium (Ca), potassium (K), magnesium (Mg), iron (Fe), and zinc (Zn). According to our previous study [14], the mineral elements present in triticale grains were Ca, K, P, Mn, Zn, S, Fe, and Cu. Compared to wheat, triticale contains higher levels of iron and zinc, making it an important crop for combating mineral deficiencies, particularly in regions where micronutrient deficiencies are prevalent [15].

Previous studies have shown that this cereal can be used in human nutrition: bakery products, pastry–confectionery products, pasta, biscuits, waffles, cereal bars, tortillas, pizza dough, breakfast cereals, dairy products, malt, unmalted grain for beer, soft drinks, spirits, biodegradable and edible packaging, etc. [4,16,17].

The evaluation of the composition of whole-grain flour and its effect on the quality and functional and technological properties of the flour are the most important stages in obtaining quality bakery products. On the other hand, the use of new raw materials in recipes for manufacturing bakery products must also take into account the consumer attitude towards these products and their acceptance on the consumer market [18]. According to our previous study [4], the sensory characteristic of bread depends on triticale varieties being very well appreciated for most of these, making triticale a promising raw material for food products. In addition to its high nutritional value, the development of whole-grain triticale flour bakery products is also due to the health benefits of these functional products recommended for their antioxidant, anticancer, and cardiovascular disease prevention properties [19,20,21]. In general, triticale grains help control diabetes, improve gastrointestinal health, stimulate circulation, increase cell production, and stimulate bone growth [22,23]. Few studies are available on the nutritional characteristics of triticale grains, and almost no data are available on the mineral and bioactive compounds of triticale. The objective of this study was to determine the physicochemical composition of different triticale varieties such as protein, wet gluten, fat, ash, moisture, carbohydrates, test weight, and thousand-kernel mass; mineral elements Ca, Na, Zn, Fe, and Cu; and total phenolic content (TPC), total flavonoid content (TFC), free radical scavenging activity (DPPH assay), and phenolic profile. Considering that the determination of bioactive compounds during extraction from triticale grains can be influenced by different factors such as temperature, our study analyzed its impact on triticale grains. This is the first study to establish the TPC, TFC, DPPH assay, and phenolic compounds such as 4-hydroxybenzoic acid, vanillic acid, caffeic acid, chlorogenic acid, p-coumaric acid, and rosmarinic acid as a function of temperature in order to obtain the best extraction value. These, along with other physicochemical and mineral element evaluations from different triticale varieties cultivated in the Republic of Moldova, provide valuable information on the nutritional profile of these grains for science, consumers, and industrial food producers.

## 2. Results

### 2.1. Physicochemical Values of Triticale Flours

Physicochemical characteristics of triticale flour samples are shown in Table 1. As may be seen, the moisture values for triticale flours were less than 12.25% for all samples, which indicates that they can present with higher stability during storage [4]. The ash value varied between 1.53 and 1.78% for triticale varieties, these data being in agreement with those reported by others [9,24]. This value is an indicator of flour mineral content. According to our data, Ingen 93 presented the highest mineral content whereas Fanica presented the lowest. Among triticale varieties, the protein content varied between 13.08 and 14.78% depending on genotype, which agrees with the values reported by others [7]. In general, the gluten content did not require high values in order to obtain bakery products of good quality, probably due to the fact that compared to wheat it presents a lower amount of protein in the endosperm [24]. The fat content varied between 1.05 and 1.65%, which is within the range reported by other researchers [7]. The carbohydrate content represents over 71% of the total chemical content of triticale flour, being the major component of it. Test weight and thousand-kernel mass show significant differences (*p* < 0.05) between the triticale samples, probably due to their size and composition variation.

### 2.2. Fourier Transform Infrared Spectroscopy (FT-IR) Analysis

**Figure 1:**
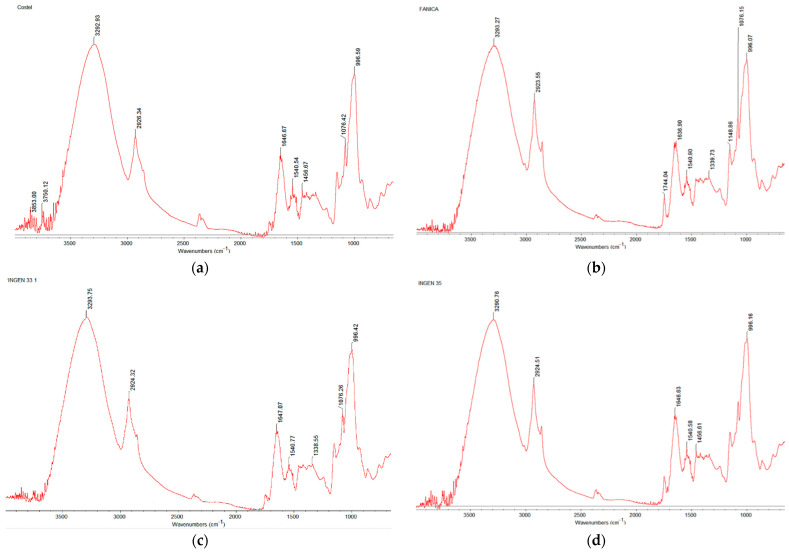

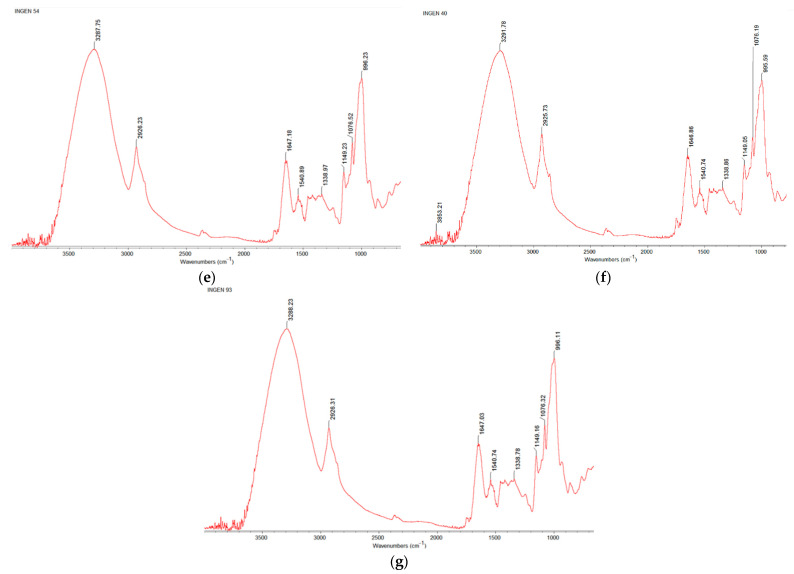
Fourier transform infrared spectroscopic (FT-IR) spectra of triticale varieties: (**a**) Costel; (**b**) Fanica; (**c**) Ingen 33; (**d**) Ingen 35; (**e**) Ingen 54; (**f**) Ingen 40; (**g**) Ingen 93.

The spectra of the triticale varieties obtained using FT-IR are shown in Figure 1. Several peaks can be seen in the spectral range (4000–800 cm^−1^), which correspond to the triticale flour compounds. FT-IR spectra obtained for the evaluated samples shows a similar spectral pattern for each of the triticale varieties, indicating similarity in terms of chemical composition. Thus, the spectral region 4000–2500 cm^−1^ corresponds to C-H stretching vibrations being considered for organic compounds, sugars, and starches; the spectral region 2500–2000 cm^−1^ corresponds to C=C and N-H or O-H stretching vibrations; and the spectral region 2000–1500 cm^−1^ corresponds to C=O stretching (carbonyl groups) or N-H bending vibrations. Peaks were also observed in the regions 800–1450 cm^−1^ (fingerprint region), which is highly specific to different types of chemical bonds and is used for identifying molecular structures. Different bending vibrations from bonds like C-H, C-C, and C-O stretching vibrations are present in the spectral region 1000–800 cm^−1^.

### 2.3. Evaluation of Total Phenolic Content (TPC), Total Flavonoid Content (TFC), and DPPH Assay

TPC, TFC, and DPPH were evaluated depending on the extraction temperature. As can be seen from Table 2, these values increased with the increase in temperature. At a temperature of 20 °C, the lowest values for TPC and TFC were recorded for the Ingen 35 variety whereas the highest was recorded for the Costel variety. Also, the Costel variety obtained the highest values for TPC and TFC at a temperature of 40 °C whereas the lowest ones were obtained for the Ingen 33 variety. The highest values of TPC and TFC were obtained for the Ingen 54 variety at a temperature of 60 °C whereas the lowest ones were for the Fanica and, respectively, Ingen 35 varieties. Our data for TPC values were lower than those reported by other researchers [24] but more similar to those reported by other studies [25]. For all extraction temperatures, the highest DPPH values were obtained for the Costel variety whereas the lowest ones were for the Fanica variety. From all triticale varieties, it seems that Costel presents the highest antioxidant properties.

### 2.4. Individual Phenolic Compounds of Triticale Samples

Individual polyphenol content of triticale varieties was identified as follows: 4-hydroxybenzoic acid, vanillic acid, caffeic acid, chlorogenic acid, *p*-coumaric acid, and rosmarinic acid. The effect of the increasing of the extraction temperature on individual polyphenol compounds was also determined because it affects the efficiency of polyphenol extraction as may be been from Table 3. Some of the phenolic compounds may undergo thermal degradation at high temperatures, reducing their concentration and making them undetectable, and others might not be efficiently extracted at low temperature, leading to lower concentrations and potential non-detection. At a temperature of 20 °C, the 4-hydroxybenzoic acid content varied between 0.45 and 1.98 mg/kg, the vanillic acid content varied between 0.42 and 1.38 mg/kg, the caffeic acid content varied between 0.17 and 0.32 mg/kg, the chlorogenic acid content varied between 0.06 and 0.17 mg/kg, the *p*-coumaric acid content varied between 9.00 and 15.12 mg/kg, and the rosmarinic acid content varied between 0.13 and 1.20 mg/kg. In general, the increase in extraction temperature led to an increase in individual polyphenolic compounds. At a temperature of 40 °C, the 4-hydroxybenzoic acid content varied between 1.10 and 3.00 mg/kg, the vanillic acid content varied between 0.76 and 1.22 mg/kg, the caffeic acid content varied between 0.15 and 0.74 mg/kg, the chlorogenic acid content varied between 0.08 and 0.40 mg/kg, the *p*-coumaric acid content varied between 11.47 and 14.56 mg/kg, and the rosmarinic acid content varied between 0.37 and 1.03 mg/kg. At a temperature of 60 °C, the 4-hydroxybenzoic acid content varied between 1.20 and 3.22 mg/kg, the vanillic acid content varied between 0.70 and 1.70 mg/kg, the caffeic acid content varied between 0.15 and 2.27 mg/kg, the chlorogenic acid content varied between 0.08 and 0.40 mg/kg, the *p*-coumaric acid content varied between 11.26 and 18.22 mg/kg, and the rosmarinic acid content varied between 0.61 and 2.67 mg/kg.

### 2.5. Mineral Content of Triticale Samples

From the point of view of the mineral elements content, the following elements were determined: Ca, Zn, Fe, Cu, and Na which are shown in Table 4. Their analysis was carried out considering the experiments previously carried out [14], which established that triticale flour has the following mineral substances: Ca, K, P, Mn, Zn, S, Fe, and Cu [14]. Through atomic absorption spectrometry, we could only identify Ca, Zn, Fe, and Cu due to the fact that we have cathode lamps for those in our faculty. We also determined Na due to the fact that it is a very important element nowadays for consumers who are aware of its impact on nutrition. Among all the analyzed mineral elements, copper was in the lowest amount whereas potassium was in the largest amount followed by phosphorus and calcium in all triticale flour samples. According to the data obtained, high variability was obtained among triticale varieties. The Ca content varied between 188 and 224 mg/kg, the Zn content varied between 11.18 and 11.68 mg/kg, the Na content varied between 19.42 and 30.49 mg/kg, the Fe content varied between 26.02 and 31.86 mg/kg, and the Cu content varied between 3.27 and 4.52 mg/kg. According to our data, Ca and Zn presented lower values than those reported by Zhu et al. [9] whereas Na and Fe presented higher values.

### 2.6. Relationships Between Physicochemical Values of Triticale Samples

**Figure 2:**
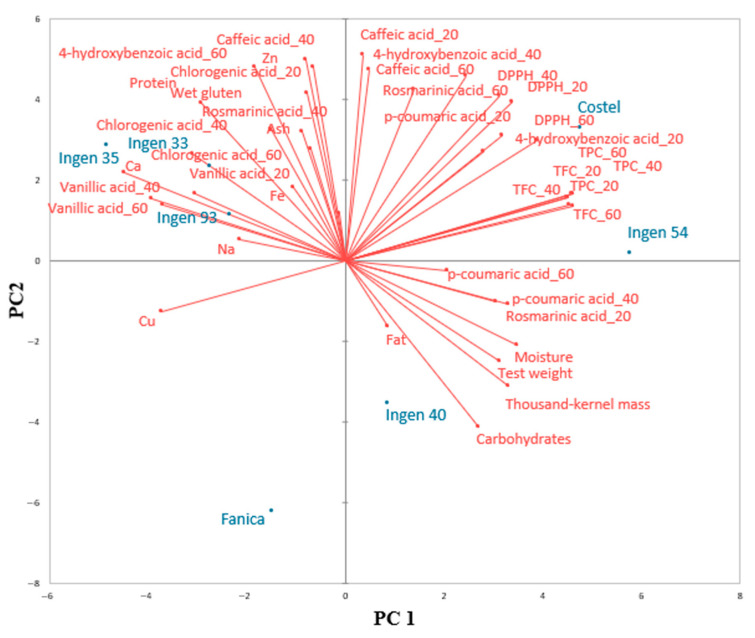
Principal component analysis of physicochemical characteristics and their relationship with triticale flours.

A PCA graph of the triticale flours related to their physicochemical characteristics is shown in Figure 2. The two axes, PC1 and PC2, represent 27.46% and 34.02% of the total variance. All the wheat grain species are closely associated with the PCA graph. Along the PCC axes, Costel and Ingen 54 are mostly related to TPC, TFC, and DPPH assay values. Ingen 40 is more related to carbohydrate content, fat value, and physical characteristics thousand-kernel mass and test weight. The triticale varieties Ingen 33, Ingen 35, and Ingen 93 are closely related to protein, gluten content, ash, vanillic acid, chlorogenic acid, and mineral elements Na, Ca, Fe, and Zn.

**Figure 3:**
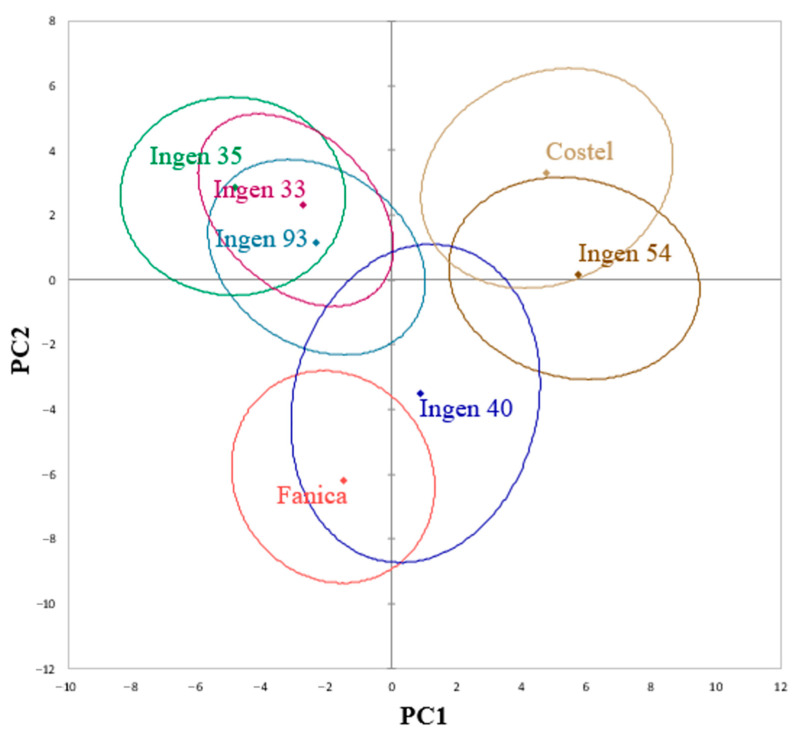
Principal component analysis of triticale flour cultivars.

A PCA plot for triticale varieties positioned by function of their physicochemical characteristics data is shown in Figure 3. The total variance in the PC axes was 61.47%, of which PC1 accounts for 34.02% and PC2 for 27.46%. As can be seen, Costel and Ingen 54 are closely related to each other, being placed on the left top side of the graph, whereas Ingen 35, Ingen 33, and Ingen 93 are placed on the right top side. Along the PC1 axis, Fanica and Ingen 40 are the mostly related to each other, whereas both PC axes clearly distinguish Fanica by Costel and Ingen 54 varieties.

## 3. Discussion

The relevance of the triticale varieties’ differences may be seen by physicochemical variation values for ash, protein, wet gluten, lipid, and carbohydrate content in the triticale samples analyzed in this research. At the same time, it was noticed that moisture content values for triticale samples vary insignificantly, which can be explained by the fact that the main factors that can influence the moisture content of cereal products are the relative humidity of the air and the variation in temperature, and since the samples were stored under the same conditions, water transfer and humidity variation occurred the same in all samples [4]. Analysis of the physicochemical properties of cereals is necessary to establish the possibility of using them in different domains of the food industry, some varieties having properties suitable for processing into flour and use in baking, pasta making, or other flour products; other varieties of cereals having low technological properties can only be used as animal feed. According to the data obtained, there are significant differences between triticale varieties (*p* < 0.05) in wet gluten content. Thus, it was found that the Ingen 33, Ingen 35, and Costel varieties are characterized by a wet gluten content greater than 25%, which means that flour from these triticale varieties can be used for the manufacture of bakery products, the minimum admissible content of gluten for baking flour being 22%. Moreover, the data obtained recommend the use of triticale flour in biscuits, cookies, or other products that need less gluten to obtain products of good quality. However, its uses in breadmaking may be sustainable for some triticale varieties with a higher gluten content such as Ingen 33, Ingen 35 or by the use or some ingredients as vital gluten in bread recipe. These findings emphasize the importance of specific triticale varieties for flour quality and baking performance. In terms of protein content, the quantity of gluten has a major impact on bakery product quality. Flour with high gluten content has good qualities for breadmaking [4,26]. Some triticale varieties had higher ash and lipid contents, which may be attributed to a higher share of seed coat and the amounts of these nutrients in the outer layers and germ of the kernel [27]. This is mainly influenced by triticale variety, as fat is primarily located in the germ and bran. From all the triticale samples analyzed, four varieties did not present significant differences (*p* < 0.05) between test weigh values, whereas the rest of the samples presented higher values for this parameter. From a technological point of view, this parameter is an important one, indicating the triticale’s potential for milling [28]. A higher hectoliter mass is characteristic of denser and fuller grains, which results in a higher flour extraction yield. Thus, the triticale varieties Ingen 54 and Fanica can be considered more suitable for flour manufacturing compared to other analyzed triticale varieties. In terms of other physical characteristics, the thousand-kernel mass is well correlated with flour yield, millers preferring grains with a higher value of this parameter [29].

Analyzing the FT-IR spectra obtained for triticale varieties, it can be observed that no significant variations were obtained among the analyzed samples. Relevant peak groups were around 1000 cm^−1^, 1100 cm^−1^, 1450 cm^−1^, 1540 cm^−1^, 1650 cm^−1^, 2900 cm^−1^, 3300 cm^−1^, 3750, and 3850 cm^−1^, which correspond to different vibrational modes of molecules, their functional groups being characteristics of different carbohydrate compounds and the lipid, protein, and moisture content of triticale flours [26]. Peaks for starch can be seen centered around 3025–4000 cm^−1^ on the basis of O-H stretching vibration but also in the 800–1400 cm^−1^ region (fingerprint region) according to Amir et al. [30]. Lipids can be identified around the 2900 cm^−1^ value due to the stretching vibration modes of the C–H bond in alkyl –CH_2_ and –CH_3_ groups [31] whereas protein can be identified around 1450 cm^−1^, 1540 cm^−1^, and 1650 cm^−1^ due to the vibration modes of the amino acids’ groups from their content. Peaks for water can be identified in the range of 3300 cm ^−1^ due to the stretching vibration of functional group OH and H. However, water quantification can be difficult to achieve, due to the spectral interference with other OH groups from the alcohol, phenol, and hydroperoxide structures, which can also present hydrogen bonding [32]. The spectral regions corresponding to the protein compounds of triticale flour are related to amide II and amide I groups situated to a wave numbers around 1540 cm^−1^ (N-H, C-N) and 1645 cm^−1^ (C=O, C-N), respectively [31]. Amide II is also specific to peptides, being more complex than amide I due to the fact that the absorption band of proteins is produced mainly by the NH bond, and secondarily by the CN bond [30]. The third peak visible around approximately 1450 cm^−1^ corresponds to amide III and is produced by the NH, CH, and CN bonds [31].

The evaluation of biologically active compounds from triticale flour was carried out by determining antioxidant activity, TPC, TFC, and individual polyphenolic compounds. In the current study, a DPPH assay was used to estimate the antioxidant activity of the triticale cultivars. Antioxidant activity can be monitored by a variety of techniques with different mechanisms, the most commonly used being the DPPH free radical scavenging method, which relies on electron donation by antioxidants to neutralize the DPPH radical. The antioxidant activity of the analyzed varieties increased with increasing temperature during extraction, with all experimental variants presenting satisfactory antioxidant activity values. The DPPH assay value for triticale samples varied between 30.48 and 70.23%, were higher than those reported by Jańczak-Pieniążek et al. [33] for triticale flours, and higher than those reported for different wheat cultivars by other researchers [34,35]. However, there was high variability between samples, which were significantly (*p* < 0.05) influenced by the triticale cultivars. The distribution of antioxidant compounds in the mass of the triticale grain was not uniform, being concentrated in the aleurone layer and the coat. As a result, their amount in triticale flour depends on its type. The lowest DPPH assay was obtained for the Fanica variety, which also had the lowest ash content, which may indicate that this cultivar contains a lower amount of coating. Tocopherols, flavonoids, and phenolic acids are the most important natural antioxidant compounds. TFC presents a similar trend to antioxidant activity for triticale cultivars. This may be due to the fact that flavonoid compounds are responsible for antioxidant activity and are considered to have a positive effect on various diseases such as cancer, Alzheimer’s disease, and atherosclerosis. Generally, flavonoids and phenolic acids are found in larger amounts in grains, and their amounts are determined by the variety, type, and part of the grain where they are located [36]. Polyphenols led to grains high in antioxidant activity [37]. Total polyphenol concentration is mostly quantified using the mg equivalent of gallic acid, one of the simplest polyphenols—although other polyphenols may be used depending on the major phenolic present in the analyzed sample [38]. Fras et al. [24] reported that the TPC value of triticale flour is similar to that of wheat flour. They reported values from 0.7 to 1.1 mg of GAE/g for triticale flour, which are generally higher compared to our obtained data. However, compared to the data reported by Lachman et al. [39] for different wheat accessions (502–686 mg GAE/kg), our data were similar for some triticale varieties, higher for others, or lower for Fanica, Ingen 35, and Ingen 33 varieties. Our TPC values obtained for triticale samples were higher than those reported by Vahler at al. [40] for different wheat varieties in grains (168–459 mg GAE/kg) or flour (44–140 mg GAE/kg).

Triticale contains numerous phenolic compounds, including 4-hydroxybenzoic acid, vanillic acid, caffeic acid, chlorogenic acid, *p*-coumaric acid, and rosmarinic acid, determined in the present study, which are known antioxidants. In grains, these phenolic acids are found both in free form and esterified with acids, sugars, and polysaccharides. Phenolic compounds inhibit lipid oxidation by scavenging free radicals that form low-energy phenolic radicals, whose energy is insufficient to oxidize lipids at significant biological rates. The comparison of our results with the existing literature on phenolic acid concentrations in triticale grains is challenging due to insufficient reporting of these concentrations. Variations in reported results may stem from the use of different analytical methods. Alijošius et al. [41] reported a mean concentration of 1.74 µg/g, 3.91 µg/g, and 11.52 µg/g in a triticale variety for *p*-hydroxybenzoic acid, vanillic acid, and *p*-coumaric acid, respectively, whereas in Weidner et al. [42], a concentration range of 0.67–3.43 µg/g and 1.08–3.37 µg/g in three triticale caryopses varieties for caffeic acid and *p*-coumaric acid, respectively. Hosseinian and Mazza [12] found vanillic acid, ferulic acid, *m*-coumaric acid, and *p*-coumaric acid in the bran, flakes, straw, and leaves of triticale. Generally, the phenolic compounds of the analyzed triticale samples increased with increasing temperature during extraction, these data being in agreement with those reported by others [43]. Moreover, in some varieties such as Fanica, Ingen 33, and Ingen 54, some of the phenolic compounds are not detected at low extraction temperature. This may be due to the impact of thermal energy on the efficiency of polyphenol extraction from triticale grains [44]. By thermal energy increase, the cellular structure of triticale is disrupted, which enhances the permeability of the cell membrane and facilitates the release of secondary metabolites, such as polyphenols. This disruption promotes solubility and the mass transfer of polyphenols by reducing surface tension, improving the wetting of the plant material, and enabling better solvent penetration. Additionally, elevated temperatures decrease the viscosity of the extraction medium, further accelerating the extraction process. Our data are in agreement with other studies, confirming the positive effect of temperature on antioxidant compound extraction across various matrices [43,45].

In terms of mineral content, the highest value of Ca was obtained for Ingen 93, followed by Ingen 33, which was significantly different (*p* < 0.05) to the rest of the triticale samples. This variation may depend on the age of the grain variety and its bran content, in relation to the grain yield [46]. Also, Ingen 93 and Ingen 33 presented the highest Zn and Fe contents, which indicates that these varieties are the most recommended one to be used for food products, especially in developing countries were many women and children have deficiencies [47]. Moreover, one-third of the world’s population is deficient in these minerals [48], and therefore their content in triticale grains is very important. Iron deficiency causes anemia, whereas zinc supports development in childhood, adolescence, pregnancy, and of the immune system [49]. According to Golea et al. [27], mineral elements are positively correlated to protein content. The causes of the correlation are not known, but it is assumed that mineral concentrations in the outer layers of triticale kernels are significantly higher than in the starchy endosperm. Variations in mineral content across different triticale cultivars can be attributed to differences in kernel size and plumpness, which affect the ratio of endosperm to total grain weight. According to our data, Na and Fe presented higher values than those reported by different researchers [9,50], whereas Ca and Zn showed lower values. From all the mineral elements determined, Cu was in the lowest amount, these data being in agreement with those reported by Biel et al. [50]. Deficiency in Cu is rare and may lead to hypochromic anemia, neutropenia, leucopenia, normocytic anemia, and osteoporosis in children.

Principal component analysis (PCA) does not capture all the variations present in the dataset. This indicates that there are other factors or characteristics that are not included in the PCA but still play a role in differentiating the triticale varieties, like environmental conditions, genetic differences, or others. The results of the principal component analysis (PCA) showed that the quality of triticale varieties was not similar, some of them being distinguished by the PC axes. However, some varieties were placed on the same right side in the PCA graphs, indicating high similarities between them. The quality of Ingen 33, Ingen 35, and Ingen 93 was more related to their chemical characteristics: gluten, protein, ash, and mineral elements Na, Ca, Fe, and Zn. These correlations are predictable, since these varieties presented the highest amount of protein, which is well correlated with gluten due to the fact that it represents a high amount of triticale protein content [27]. Also, the close association between ash and most of the mineral elements determined is because they are part of ash, which represents the total mineral content of triticale samples [26]. The closeness of Ingen 40 to carbohydrates, thousand-kernel mass, and test weight is due to the fact that this variety presents the highest amount of carbohydrate and also due to the fact that test weight and thousand-kernel mass are closely associated according to the data reported by others [27]. According to the biologically active compounds, it seems that they are mostly related to the Ingen 54 and Costel varieties and less to the Ingen 40 one. However, Ingen 33, Ingen 35, and Ingen 93 were well associated with vanillic acid, chlorogenic acid, caffeic acid, 4-hydroxibenzoic acid, and rosmarinic acid at extraction temperatures of 40 and 60 °C. According to the PCA graph, Fanica is differently positioned to the rest of the samples. This may be due to the fact that this sample has a combination of feature values that is quite different from others, which could cause that sample to make a large contribution to the first few principal components. According to the data obtained, the Fanica sample had the fewest phenolic compounds detected from all triticale varieties. Also, it presented the lowest DPPH values and the highest moisture content. This made the Fanica sample stand out in the lower-dimensional representation. A comparison of our correlation data with the existing literature is difficult to make since there are insufficient reports on the concentrations of biologically active compounds in triticale grains.

## 4. Materials and Methods

### 4.1. Triticale Flour Samples Physicochemical Characteristics

Seven triticale grain varieties, namely, Ingen 35, Ingen 33, Costel, Fanica, Ingen 54, Ingen 40, and Ingen 93, cultivated in the Republic of Moldova (harvest 2023) were used. The triticale varieties were provided by the Institute of Genetics, Physiology, and Plant Protection from Republic of Moldova. The grains were milled into flour by using a laboratory mill 3100 (Perten Instruments, Hägersten, Sweden), and the following characteristics were determined according to ICC methods: protein content (105/2), wet gluten content (137/1), ash content (104/1), lipid content (ICC 136), and moisture (ICC 110/1). Carbohydrate content was determined through differences according to Golea et al. [26]. Thousand-kernel mass was determined according to ISO 520:2010 [51] and test weight according to ISO 7971-1:2009 method [52].

### 4.2. FT-IR Analysis

To analyze the composition changes in triticale flours, FT-IR with a Nicolet iS-20 spectrophotometer (Thermo Scientific, Karlsruhe, Dieselstraβe, Germany) in attenuated total reflectance (ATR) mode was used. For that purpose, a resolution of 4 cm^−1^ was applied in the wave number range from 4000 to 650 cm^−1^. For FT-IR spectra analysis, a sample of 2 g of triticale flour was pressed and the pellet obtained was placed on the ATR crystal.

### 4.3. Determination of Bioactive Compounds from Triticale Flours

The bioactive compound extraction technique required the mixing 1 g of triticale flour with 5 mL of 40% methanol, which was stirred for 15 min at three different temperatures: 20 °C, 40 °C, and 60 °C. After extraction, the samples were filtered and stored at −20 °C for further analysis. TPC, TFC, and antioxidant activity were determined according to the method described by Procopet et al. [53]. For TPC, TFC, and DPPH, a spectrophotometer was used to measure absorbance at 750 nm, 425 nm, and 518 nm, respectively. The TPC data obtained were expressed in mg of gallic acid equivalent (GAE)/kg whereas the TFC was expressed in mg of quercetin equivalent (QE)/kg. The phenolic acids (4-hydroxybenzoic acid, vanillic acid, caffeic acid, chlorogenic acid, *p*-coumaric acid, and rosmarinic acid) were determined using a Shimadzu high-performance liquid chromatograph (Kyoto, Japan) with a diode array detector coupled with a DAD detector. The separation of the compounds was performed with a Kinetex 2.6 μm Biphenyl 100 Å, LC Column 150 × 4.6 mm chromatographic column (Phenomenex, Torrance, California, USA) at 25 °C. The flow rate was 0.5 mL/min and the injection volume 20 μL. The analysis method used was that described by Oroian et al. [43]. The identification of the individual polyphenols in the samples was performed at a wavelength of 280 nm for vanillic acid and *p*-hydroxybenzoic acid and at a wavelength of 320 nm for chlorogenic acid, caffeic acid, *p*-coumaric acid, and rosmarinic acid.

### 4.4. Mineral Elements of Triticale Flours

Mineral elements of triticale flours Ca, Na, Fe, Zn, and Cu were determined by using flame atomic absorption spectrometry with an AA-6300 Shimadzu spectrometer. For that propose, 10 g of triticale flour was calcined at a temperature of up to 450 °C for 8 h. The ash obtained was digested with 10 mL of 0.1 mol/L nitric acid (HNO_3_) on a hot plate, and the volume was then adjusted to 50 mL with deionized water. Standard solutions of Ca, Na, Fe, Zn, and Cu (Sigma-Aldrich/Merck, Darmstadt, Germany) were used for calibration. To prevent contamination, all glassware was thoroughly cleaned with HNO_3_ solution and rinsed with deionized water after each use.

### 4.5. Statistical Analysis

Analysis of variance (ANOVA) was used to analyze the data, with Tukey’s test at a significance level of α = 0.05. The data analysis was made using Statgraphics Centurion XVI 16.1.17 software (Statgraphics Technologies, Inc., The Plains, VA, USA). Principal component analysis (PCA) was performed using XLSTAT 2021.2.1 software (Addinsoft, New York, NY, USA).

## 5. Conclusions

There is high variability in physicochemical composition between triticale grain varieties. The moisture values were lower than 12.25% for all triticale varieties, indicating the long shelf life of the grains. All triticale grains can be used to produce bakery products, their composition being like that of wheat for some cultivars. The FT-IR spectra showed no significant differences among the triticale varieties. Relevant peak groups were seen around 1000 cm^−1^, 1100 cm^−1^, 1450 cm^−1^, 1540 cm^−1^, 1650 cm^−1^, 2900 cm^−1^, 3300 cm^−1^, 3750 cm^−1^, and 3850 cm^−1^, corresponding to different triticale compounds such as carbohydrates, lipids, proteins, and moisture content in triticale flours. The highest calcium (Ca) content was found in Ingen 93, followed by Ingen 33, with significant differences (*p* < 0.05) compared to other triticale varieties. Ingen 93 and Ingen 33 also exhibited the highest zinc (Zn) and iron (Fe) amount, which making their use in food products recommendable. Free radical scavenging activity (DPPH assay) of triticale cultivars ranged from 30.48% to 76.64%, being higher than those reported by other triticale and wheat cultivars. Total flavonoid and phenolic content exhibited a similar trend to antioxidant activity, indicating that triticale grains may be a valuable source of polyphenolic and flavonoid compounds, contributing to antioxidant capacity. A variety of phenolic compounds were determined in triticale grain varieties, such as 4-hydroxybenzoic acid, vanillic acid, caffeic acid, chlorogenic acid, *p*-coumaric acid, and rosmarinic acid, which are known for their antioxidant properties. Some of them were not detected in triticale varieties, such as Fanica, Ingen 33, and Ingen 54, at low extraction temperatures. The highest value for phenolic acid was obtained by *p*-coumaric acid fallowed by rosmarinic acid, caffeic acid, 4-hydroxybenzoic acid, vanillic acid, and chlorogenic acid. Principal component analysis (PCA) showed that triticale varieties present differences in quality, with some varieties showing clear distinctions based on their physicochemical characteristics, while others displayed high similarities. In general, some varieties, such as Ingen 33, Ingen 35, and Ingen 93, were highly correlated with their chemical characteristics, whereas others, such as the Ingen 54 and Costel varieties, were more associated with their biologically active compounds. Due to a lack of sufficient literature on the physicochemical characteristics of triticale grains, comparisons between the current study and existing research are challenging, indicating a need for more comprehensive data on this subject.

## Figures and Tables

**Table 1:** Physicochemical parameters of triticale flour.

Samples	Moisture (%)	Ash (%)	Protein (%)	Wet Gluten (%)	Fat (%)	Carbohydrates (%)	Test Weight (kg hL^−1^)	Thousand-Kernel Mass (g)
Fanica	12.24 ± 0.01 ^f^	1.53 ± 0.00 ^a^	13.87 ± 0.06 ^b^	21.72 ± 0.16 ^c^	1.30 ± 0.02 ^b^	71.06 ± 0.05 ^e^	76.12 ± 0.10 ^c^	41.52 ± 0.03 ^e^
Ingen 93	12.03 ± 0.00 ^a^	1.78 ± 0.01 ^e^	14.56 ± 0.05 ^d^	18.68 ± 0.07 ^a^	1.34 ± 0.01 ^b^	70.29 ± 0.07 ^b^	73.42 ± 0.07 ^a^	38.24 ± 0.01 ^c^
Ingen 35	12.11 ± 0.01 ^c^	1.62 ± 0.01 ^c^	14.78 ± 0.08 ^e^	26.32 ± 0.12 ^e^	1.31 ± 0.01 ^b^	70.14 ± 0.04 ^a^	73.22 ± 0.05 ^a^	37.41 ± 0.05 ^b^
Ingen 33	12.08 ± 0.01 ^b^	1.72 ± 0.01 ^d^	14.57 ± 0.07 ^d^	27.45 ± 0.14 ^f^	1.33 ± 0.02 ^b^	70.30 ± 0.06 ^b^	73.14 ± 0.09 ^a^	37.11 ± 0.00 ^a^
Ingen 54	12.21 ± 0.01 ^e^	1.55 ± 0.01 ^b^	13.69 ± 0.02 ^b^	19.66 ± 0.06 ^b^	1.65 ± 0.02 ^d^	70.90 ± 0.05 ^d^	79.16 ± 0.05 ^d^	43.82 ± 0.04 ^f^
Ingen 40	12.16 ± 0.00 ^d^	1.71 ± 0.01 ^d^	13.08 ± 0.04 ^a^	18.51 ± 0.09 ^a^	1.44 ± 0.00 ^c^	71.61 ± 0.01 ^f^	74.62 ± 0.03 ^b^	39.68 ± 0.06 ^d^
Costel	12.25 ± 0.01 ^f^	1.73 ± 0.00 ^d^	14.18 ± 0.05 ^c^	25.63 ± 0.07 ^d^	1.05 ± 0.01 ^a^	70.79 ± 0.01 ^c^	73.54 ± 0.08 ^a^	38.57 ± 0.03 ^c^

Mean values followed by the same letter within a column are significantly different (*p* < 0.05).

**Table 2:** Total phenolic content, total flavonoid content, and DPPH assay of triticale samples.

Triticale Varieties	T (°C)	Total Phenolic Content (mg GAE/kg)	Total Flavonoid Content (mg QE/kg)	DPPH (%)
Fanica	20	268.7 ± 11.4 ^a^	144.1 ± 2.5 ^a,b^	30.48 ± 1.04 ^a^
	40	331.4 ± 13.8 ^b^	177.8 ± 3.9 ^b,c^	39.46 ± 1.18 ^b^
	60	392.5 ± 10.3 ^c^	210.6 ± 7.9 ^c,d^	43.30 ± 1.01 ^c^
Ingen 93	20	443.8 ± 11.7 ^d^	238.1 ± 9.8 ^d^	42.02 ± 0.95 ^c^
	40	515.6 ± 12.0 ^e^	266.9 ± 10.1 ^e^	48.43 ± 0.91 ^d^
	60	596.8 ± 15.7 ^f^	308.9 ± 10.7 ^f^	49.71 ± 0.27 ^e^
Ingen 35	20	227.0 ± 5.6 ^a^	117.6 ± 2.1 ^a^	45.87 ± 0.62 ^d^
	40	341.5 ± 12.1 ^b^	176.8 ± 1.7 ^b^	49.71 ± 0.28 ^e^
	60	405.8 ± 14.5 ^c^	210.0 ± 3.4 ^c^	52.28 ± 0.57 ^e^
Ingen 33	20	275.9 ± 9.3 ^a^	142.8 ± 1.9 ^a,b^	56.12 ± 0.82 ^f^
	40	306.6 ± 11.8 ^b^	158.7 ± 4.1 ^b^	57.41 ± 0.57 ^f^
	60	361.1 ± 3.9 ^c^	186.9 ± 3.8 ^c^	63.82 ± 0.66 ^g^
	20	591.6 ± 13.1 ^f^	306.5 ± 6.2 ^f^	65.10 ± 0.59 ^g^
Ingen 54	40	661.4 ± 8.6 ^g^	342.7 ± 5.4 ^g^	66.38 ± 0.14 ^g^
	60	877.1 ± 14.6 ^j^	454.5 ± 4.9 ^i^	76.64 ± 0.81 ^i^
Ingen 40	20	474.2 ± 13.5 ^d^	245.7 ± 3.6 ^d^	43.30 ± 0.93 ^c^
	40	498.0 ± 1.6 ^d^	258.1 ± 4.9 ^e^	45.46 ± 0.71 ^b^
	60	511.8 ± 7.2 ^e^	265.2 ± 6.1 ^e^	54.84 ± 0.47 ^f^
Costel	20	727.7 ± 6.8 ^h^	396.3 ± 3.0 ^h^	65.10 ± 0.61 ^g^
	40	748.9 ± 10.1 ^h^	407.9 ± 7.8 ^h^	67.66 ± 0.72 ^h^
	60	834.1 ± 11.5 ^i^	454.3 ± 8.1 ^i^	70.23 ± 0.49 ^h^

Mean values followed by the same letter within a column are significantly different (*p* < 0.05). GAE—acid gallic equivalents; QE—quercetin equivalents.

**Table 3:** Individual phenolic compound values of triticale samples.

Varieties		Phenolic Compounds (mg/kg)					
	T (°C)	4-Hydroxybenzoic Acid	Vanillic Acid	Caffeic Acid	Chlorogenic Acid	*p*-Coumaric Acid	Rosmarinic Acid
Fanica	20	-	-	-	-	9.00 ± 0.15 ^a^	0.72 ± 0.06 ^d^
	40	0.82 ± 0.01 ^b^	-	-	-	11.47 ± 0.21 ^c^	0.53 ± 0.08 ^c^
	60	1.20 ± 0.03 ^c^	0.79 ± 0.02 ^b,c^	0.15 ± 0.01 ^a^	0.17 ± 0.01 ^d^	11.26 ± 0.08 ^c^	0.97 ± 0.05 ^e^
Ingen 93	20	0.45 ± 0.02 ^a^	1.38 ± 0.02 ^g^	0.21 ± 0.02 ^b^	0.16 ± 0.01 ^c,d^	12.28 ± 0.17 ^c^	0.51 ± 0.04 ^c^
	40	2.68 ± 0.05 ^i^	1.04 ± 0.03 ^e^	0.37 ± 0.02 ^b,c^	0.12 ± 0.01 ^b,c^	11.78 ± 0.13 ^c^	0.53 ± 0.07 ^c^
	60	2.21 ± 0.03 ^g^	1.70 ± 0.01 ^h^	1.13 ± 0.02 ^f^	0.24 ± 0.01 ^e^	14.68 ± 0.18 ^e^	1.45 ± 0.05 ^g^
Ingen 35	20	0.35 ± 0.01 ^a^	1.03 ± 0.03 ^e^	0.32 ± 0.03 ^b^	0.06 ± 0.01 ^a^	12.42 ± 0.15 ^c^	0.33 ± 0.03 ^b^
	40	1.95 ± 0.02 ^f^	1.22 ± 0.02 ^f^	0.66 ± 0.03 ^d^	0.40 ± 0.02 ^g^	13.18 ± 0.02 ^d^	1.03 ± 0.04 ^e^
	60	3.22 ± 0.06 ^k^	1.25 ± 0.01 ^f^	1.72 ± 0.05 ^i^	0.40 ± 0.01 ^g^	14.22 ± 0.05 ^d^	2.60 ± 0.02 ^i^
Ingen 33	20	1.98 ± 0.01 ^f^	-	0.23 ± 0.01 ^b^	0.16 ± 0.01 ^c,d^	10.86 ± 0.12 ^b^	0.13 ± 0.01 ^a^
	40	3.00 ± 0.05 ^j^	0.76 ± 0.02 ^b^	0.58 ± 0.02 ^d^	0.14 ± 0.01 ^c^	14.56 ± 0.09 ^e^	0.53 ± 0.04 ^c^
	60	2.97 ± 0.03 ^j^	0.84 ± 0.03 ^c^	1.57 ± 0.05 ^h^	0.34 ± 0.01 ^f^	18.22 ± 0.27 ^g^	1.28 ± 0.07 ^f,g^
	20	1.68 ± 0.02 ^e^	-	0.26 ± 0.02 ^b^	-	15.12 ± 0.13 ^e^	1.20 ± 0.05 ^f^
Ingen 54	40	2.57 ± 0.01 ^h,i^	-	0.15 ± 0.01 ^a^	0.10 ± 0.01 ^b^	13.51 ± 0.20 ^d^	0.70 ± 0.04 ^d^
	60	2.84 ± 0.01 ^i^	0.72 ± 0.01 ^b^	1.95 ± 0.04 ^j^	0.10 ± 0.01 ^b^	11.87 ± 0.17 ^c^	2.25 ± 0.08 ^h^
Ingen 40	20	1.09 ± 0.02 ^c^	0.98 ± 0.01 ^d^	0.17 ± 0.01 ^a^	-	11.91 ± 0.15 ^c^	0.23 ± 0.01 ^b^
	40	1.10 ± 0.01 ^c^	1.00 ± 0.02 ^d,e^	0.27 ± 0.01 ^b^	0.08 ± 0.01 ^a,b^	12.48 ± 0.09 ^c^	0.37 ± 0.04 ^b^
	60	1.29 ± 0.04 ^d^	0.73 ± 0.01 ^b^	1.39 ± 0.03 ^g^	0.11 ± 0.01 ^b,c^	14.26 ± 0.11 ^e^	0.61 ± 0.02 ^c^
Costel	20	1.58 ± 0.03 ^e^	0.93 ± 0.02 ^d^	0.25 ± 0.02 ^b^	0.17 ± 0.01 ^d^	13.09 ± 0.24 ^d^	0.55 ± 0.03 ^c^
	40	2.35 ± 0.05 ^h^	-	0.74 ± 0.02 ^d,e^	-	14.14 ± 0.16 ^d^	0.68 ± 0.03 ^d^
	60	2.25 ± 0.03 ^g^	0.70 ± 0.01 ^a,b^	2.27 ± 0.04 ^k^	0.08 ± 0.01 ^a,b^	16.35 ± 0.12 ^f^	2.67 ± 0.07 ^i^

^a–k^, mean values (*n* = 3) in the same column followed by different letters are significantly different (*p* < 0.05).

**Table 4:** Mineral content of triticale samples.

Sample	Ca (mg/kg)	Zn (mg/kg)	Na (mg/kg)	Fe (mg/kg)	Cu (mg/kg)
Ingen 33	217.9 ± 0.2 ^d^	11.68 ± 0.04 ^e^	28.19 ± 0.14 ^d^	30.26 ± 0.05 ^c^	4.04 ± 0.01 ^d^
Ingen 35	207.9 ± 0.5 ^c^	11.59 ± 0.03 ^d^	29.38 ± 0.14 ^e^	28.41 ± 0.10 ^b^	4.11 ± 0.05 ^d^
Ingen 93	224.6 ± 0.3 ^e^	11.60 ± 0.02 ^d^	26.71 ± 0.11 ^c^	29.75 ± 0.12 ^c^	4.52 ± 0.01 ^f^
Ingen 54	172.6 ± 0.4 ^a^	11.45 ± 0.00 ^c^	26.98 ± 0.13 ^c^	27.85 ± 0.07 ^b^	3.77 ± 0.03 ^c^
Ingen 40	204.5 ± 0.6 ^c^	11.22 ± 0.06 ^b^	19.42 ± 0.11 ^a^	26.02 ± 0.09 ^a^	3.61 ± 0.06 ^b^
Fanica	188.4 ± 0.5 ^b^	11.18 ± 0.00 ^a^	30.49 ± 0.21 ^e^	31.08 ± 0.12 ^d^	4.32 ± 0.03 ^e^
Costel	188.5 ± 0.6 ^b^	11.41 ± 0.04 ^c^	24.76 ± 0.09 ^b^	31.86 ± 0.11 ^e^	3.27 ± 0.04 ^a^

^a–f^, mean values (*n* = 3) in the same column followed by different letters are significantly different (*p* < 0.05).

## Data Availability

The original contributions presented in this study are included in the article. Further inquiries can be directed to the corresponding author.

## References

[1] González-Alonso V., Pradal I., Wardhana Y.R., Cnockaert M., Wieme A.D., Vandamme P., De Vuyst L. (2024). Microbial Ecology and Metabolite Dynamics of Backslopped Triticale Sourdough Productions and the Impact of Scale. Int. J. Food Microbiol..

[2] Contreras M.d.M., Bravo F.I., Carrillo W. (2024). Potential Sources of Novel Foods to Procure Nutrients and Bioactive Compounds for Disease Prevention. Nutrients.

[3] Faccini N., Morcia C., Terzi V., Rizza F., Badeck F.-W. (2023). Triticale in Italy. Biology.

[4] Ghendov-Mosanu A., Popa N., Paiu S., Boestean O., Bulgaru V., Leatamborg S., Lupascu G., Codină G.G. (2024). Breadmaking Quality Parameters of Different Varieties of Triticale Cultivars. Foods.

[5] Kaszuba J., Jaworska G., Krochmal-Marczak B., Kogut B., Kuźniar P. (2021). Effect of bran addition on rheological properties of dough and quality of triticale bread. J. Food Process. Preserv..

[6] Nocente F., De Francesco G., Marconi O., Floridi S., Latini A., Cantale C., Cantale C., Galeffi P., Ammar K., Gazza L. (2024). Malting and brewing process optimization of elite lines of triticale for beer production. Food Bioprocess Technol..

[7] Camerlengo F., Kiszonas A.M. (2023). Genetic Factors Influencing Triticale Quality for Food. J. Cereal Sci..

[8] Kandrokov K.R. (2023). Effects of triticale flour on the quality of honey cookies. Foods Raw Mater..

[9] Zhu F. (2018). Triticale: Nutritional composition and food uses. Food Chem..

[10] Chen O., Costa S.M., Carolo K. (2019). Phenolic Acids. Whole Grains and Their Bioactives.

[11] Xing P., Song Z., Li X. (2020). Differences in the metabolite profiles of tender leaves of wheat barley, rye and triticale based on LC-MS. bioRxiv.

[12] Hosseinian F.S., Mazza G. (2009). Triticale bran and straw: Potential new sources of phenolic acids, proanthocyanidins, and 516 lignans. J. Funct. Foods.

[13] Kaszuba J., Kapusta I., Posadzka Z. (2021). Content of Phenolic Acids in the Grain of Selected Polish Triticale Cultivars and Its Products. Molecules.

[14] Ursachi F.V., Codină G.G., Atudorei D., Paiu S., Rumeus I., Lyatamborg S., Lupashku G.A., Ghendov-Moşanu A. Mineral variability of different triticale flours varieties cultivated in Republic of Moldova. Proceedings of the Life Sciences Today for Tomorrow.

[15] Skovmand B., Fox P.N., Villareal R.L., Brady N.C. (1984). Triticale in Commercial Agriculture: Progress and Promise. Advances in Agronomy.

[16] Meng X., Li T., Zhao J., Fan M., Qian H., Li Y., Wang L. (2023). Effects of different bran pretreatments on rheological and functional properties of triticale whole-wheat flour. Food Bioprocess Technol..

[17] Pribić M., Kamenko I., Despotović S., Mirosavljević M., Pejin J. (2024). Modeling and Optimization of Triticale Wort Production Using an Artificial Neural Network and a Genetic Algorithm. Foods.

[18] Gómez M., Gutkoski L.C., Bravo-Núñez Á. (2020). Understanding whole-wheat flour and its effect in breads: A review. Compr. Rev. Food Sci. Food Saf..

[19] Banu I., Patraşcu L., Vasilean I., Horincar G., Aprodu I. (2020). Impact of Germination and Fermentation on Rheological and Thermo-Mechanical Properties of Wheat and Triticale Flours. Appl. Sci..

[20] Li H., Li H., Liu Y., Liu R., Siriamornpun S. (2024). Optimization of Heat–Moisture Treatment Conditions for High-Amylose Starch and Its Application in High-Resistant Starch Triticale Noodles. Foods.

[21] Liu M., Fan M., Qian H., Li Y., Wang L. (2023). Effect of different enzymes on thermal and structural properties of gluten, gliadin, and glutenin in triticale whole-wheat dough. Int. J. Biol. Macromol..

[22] Arizmendi-Cotero D., Bernal-Estrada M.A., Dominguez-Lopez A., Díaz-Ramírez M., Ponce-García N., Villanueva-Carvajal A. (2020). Endogenous enzymes of triticale used as natural sweeteners of wheat-triticale cookies. Cereal Chem..

[23] Piazza I., Carnevali P., Faccini N., Baronchelli M., Terzi V., Morcia C., Ghizzoni R., Patrone V., Morelli L., Cervini M. (2023). Combining Native and Malted Triticale Flours in Biscuits: Nutritional and Technological Implications. Foods.

[24] Fraś A., Gołębiewska K., Gołębiewska D., Wiśniewska M., Gzowska M., Mańkowski D.R. (2021). Utilisation of triticale (X Triticosecale Wittmack) and residual oat flour in breadmaking. Czech J. Food Sci..

[25] Straumite E., Galoburda R., Tomsone L., Kruma Z., Gramatin A.I., Kronberga A., Sturite I. (2017). Nutritional quality of triticale (×Triticosecale Wittm.) grown under different cropping systems. Proc. Latv. Acad. Sci. Sect. B Nat. Exact Appl. Sci..

[26] Golea C.M., Codină G.G., Oroian M. (2023). Prediction of Wheat Flours Composition Using Fourier Transform Infrared Spectrometry (FT-IR). Food Control.

[27] Golea C.M., Stroe S.-G., Gâtlan A.-M., Codină G.G. (2023). Physicochemical Characteristics and Microstructure of Ancient and Common Wheat Grains Cultivated in Romania. Plants.

[28] Rachoń L., Bobryk-Mamczarz A., Kiełtyka-Dadasiewicz A. (2020). Hulled Wheat Productivity and Quality in Modern Agriculture Against Conventional Wheat Species. Agriculture.

[29] Longin C.F.H., Ziegler J., Schweiggert R., Koehler P., Carle R., Würschum T. (2016). Comparative Study of Hulled (Einkorn, Emmer, and Spelt) and Naked Wheats (Durum and Bread Wheat): Agronomic Performance and Quality Traits. Crop Sci..

[30] Amir R.M., Anjum F.M., Khan M.I., Khan M.R., Pasha I., Nadeem M. (2013). Application of Fourier transform infrared (FTIR) spectroscopy for the identification of wheat varieties. J. Food Sci. Technol..

[31] Arslan F.N., Akin G., Karuk Elmas S.N., Üner B., Yilmaz I., Janssen H.-G., Kenar A. (2020). FT-IR spectroscopy with chemometrics for rapid detection of wheat flour adulteration with barley flour. J. Consum. Prot. Food Saf..

[32] Dong X., Sun X. (2013). A case study of characteristic bands selection in near-infrared spectroscopy: Nondestructive detection of ash and moisture in wheat flour. J. Food Meas. Charact..

[33] Jańczak-Pieniążek M., Horvat D., Viljevac Vuletić M., Kovačević Babić M., Buczek J., Szpunar-Krok E. (2023). Antioxidant Potential and Phenolic Acid Profiles in Triticale Grain under Integrated and Conventional Cropping Systems. Agriculture.

[34] Horvat D., Šimić G., Drezner G., Lalić A., Ledenčan T., Tucak M., Plavšić H., Andrić L., Zdunić Z. (2020). Phenolic Acid Profiles and Antioxidant Activity of Major Cereal Crops. Antioxidants.

[35] Mpofu A., Sapirstein H.D., Beta T. (2006). Genotype and environmental variation in phenolic content, phenolic acid composition, and antioxidant activity of hard spring wheat. J. Agric. Food Chem..

[36] Žilić S. (2016). Phenolic compounds of wheat their content, antioxidant capacity and bioaccessibility. MOJ Food Process Technol..

[37] Pauliuc D., Dranca F., Oroian M. (2020). Antioxidant Activity, Total Phenolic Content, Individual Phenolics and Physicochemical Parameters Suitability for Romanian Honey Authentication. Foods.

[38] Singh J.P., Kaur A., Singh N., Nim L., Shevkani K., Kaur H., Arora D.S. (2015). In vitro antioxidant and antimicrobial properties of jambolan (*Syzygium cumini*) fruit polyphenols. LWT Food Sci. Technol..

[39] Lachman J., Musilová J., Kotíková Z., Hejtmánková K., Orsák M., Přibyl J. (2012). Spring, einkorn and emmer wheat species—Potential rich sources of free ferulic acid and other phenolic compounds. Plant Soil Environ..

[40] Vaher M., Matso K., Levandi T., Helmja K., Kaljurand M. (2010). Phenolic compounds and the antioxidant activity of the bran, flour and whole grain of different wheat varieties. Procedia Chem..

[41] Alijošius S., Šasyte V., Mieželienė A., Alenčikienė G., Bliznikas S., Racevičiūtė-Stupelienė A., Nutautaitė M., Paleckaitis M. (2018). Effect of triticale and non-starch polysaccharides (NSP) degrading enzymes on color and sensory characteristics of broiler meat. Vet. Med. Zoot..

[42] Weidner S., Amarowicz R., Karamać M., Dąbrowski G. (1999). Phenolic acids in caryopses of two cultivars of wheat, rye and triticale that display different resistance to pre-harvest sprouting. Eur. Food Res. Technol..

[43] Oroian M., Ursachi F., Dranca F. (2020). Influence of ultrasonic amplitude, temperature, time and solvent concentration on bioactive compounds extraction from propolis. Ultrason. Sonochem..

[44] Mustafa A., Turner C. (2011). Pressurized liquid extraction as a green approach in food and herbal plants extraction: A review. Anal. Chim. Acta.

[45] Dranca F., Oroian M. (2017). Total monomeric anthocyanin, total phenolic content and antioxidant activity of extracts from eggplant (*Solanum melongena* L.) peel using ultrasonic treatments. J. Food Process Eng..

[46] Simsek S., Budak B., Schwebach C.S., Ovando-Martínez M. (2019). Historical vs. Modern Hard Red Spring Wheat: Analysis of the Chemical Composition. Cereal Chem..

[47] Zhao F.J., Su Y.H., Dunham S.J., Rakszegi M., Bedo Z., McGrath S.P., Shewry P.R. (2009). Variation in Mineral Micronutrient Concentrations in Grain of Wheat Lines of Diverse Origin. J. Cereal Sci..

[48] Golea M.C., ¸Sandru M.D., Codină G.G. (2022). Mineral composition of flours produced from modern and ancient wheat varieties cultivated in Romania. Ukr. Food J..

[49] Ertop M.H., Bektaş M., Atasoy R. (2020). Effect of cereals milling on the contents of phytic acid and digestibility of minerals and protein. Ukr. Food J..

[50] Biel W., Jaroszewska A., Stankowski S., Sobolewska M., Kępińska-Pacelik J. (2021). Comparison of Yield, Chemical Composition and Farinograph Properties of Common and Ancient Wheat Grains. Eur. Food Res. Technol..

[51] (2010). International Organization for Standardization (ISO 2010). Cereals and Pulses-Determination of the Mass of 1000 Grains.

[52] (2009). International Organization for Standardization (ISO 2009). Determination of Bulk Density, Called Mass per Hectolitre-Part 1: Reference Method.

[53] Procopet O., Oroian M. (2022). Amaranth Seed Polyphenol, Fatty Acid and Amino Acid Profile. Appl. Sci..

